# Evaluating a Behavioral Insights–Informed Social Media Campaign to Increase HPV Vaccination During Routine Immunization in Nigeria

**DOI:** 10.3390/vaccines14040328

**Published:** 2026-04-07

**Authors:** Sohail Agha, Ifeanyi Nsofor, Wu Zeng

**Affiliations:** 1 Department of Epidemiology, University of Washington, Seattle, WA 98195, USA; 2 Behavioral Insights Lab, Seattle, WA 98040, USA; 3 Centre for Family Health Initiative, Abuja 902101, Nigeria; i.nsofor@atlanticfellows.org; 4 Department of Global Health, Georgetown University, Washington, DC 20057, USA; wz192@georgetown.edu; 5 Heller School for Social Policy & Management, Brandeis University, Waltham, MA 02453, USA

**Keywords:** HPV vaccination, social media, behavioral insights, motivation, ability, Fogg Behavior Model, Nigeria, difference-in-difference, vaccine uptake

## Abstract

**Background**: Cervical cancer remains a leading cause of cancer-related deaths among women in Nigeria. In 2023, the Government of Nigeria, with support from Gavi and partners, introduced the single-dose human papillomavirus (HPV) vaccine through a phased, school-based campaign. The first phase was launched in October 2023 across 16 states, followed by a second phase in May 2024 that expanded coverage to the remaining states and the Federal Capital Territory. This study evaluates the additional impact of a behavioral insights–informed digital intervention, comprising a social media campaign amplified by trained pharmacists serving as local influencers, implemented in 2025 to increase acceptance and uptake of HPV vaccination during routine immunization. **Methods**: A pre-test/post-test quasi-experimental design with a control group was implemented in three Nigerian states in 2025 to assess the additional impact of a behavioral insights–informed social media campaign designed to strengthen social approval for HPV vaccination, increase awareness of vaccination locations, and reinforce caregivers’ recognition of their adolescent daughters’ desire to be vaccinated. Messages were amplified by trained pharmacists who served as local influencers. Caregivers of adolescent girls aged 9–17 years were recruited online through targeted Facebook and Instagram advertisements during Nigeria’s transition from school-based HPV vaccination campaigns to routine immunization. Caregivers in treatment areas were exposed to geofenced social media advertisements on Facebook and Instagram and pharmacist counseling, while those in control areas were not. Logistic regression models using a difference-in-difference approach estimated the campaign’s effect on HPV vaccination, controlling for caregiver and adolescent characteristics. Additional statistical models assessed the campaign’s impact on caregivers’ motivation and ability—key drivers of behavior according to the Fogg Behavior Model. **Results**: HPV vaccination increased at a significantly higher rate in the treatment compared to the control area. The adjusted odds of an adolescent girl being vaccinated were 1.48 times higher in the treatment area at follow-up (95% CI: 1.14–1.92). Adjusted marginal effects indicated that exposure to the campaign increased the probability of vaccination by 8.9 percentage points relative to the control group. The rate at which caregivers’ motivation (aOR = 1.31, 95% CI: 1.00–1.70) and ability (knowing where to get vaccinated: aOR = 1.38, 95% CI: 1.07–1.79; ease of vaccination: aOR = 1.59, 95% CI: 1.22–2.06) increased was also higher in the treatment area. There was no relative increase in intervention versus control groups in factual knowledge regarding HPV vaccination. **Conclusions**: A behavioral insights–informed social media campaign in which pharmacists served as influencers was associated with higher HPV vaccine uptake during routine immunization. The higher rate of vaccination observed in intervention areas was associated with higher rates of caregiver motivation and ability but not with higher rates of caregiver knowledge. These findings are consistent with the potential of behavioral insights–informed digital campaigns to complement routine immunization efforts and improve vaccine uptake in low- and middle-income countries.

## 1. Introduction

Public health interventions informed by behavioral science are widely regarded as more effective at producing sustained behavior change than approaches that do not explicitly draw on behavioral theory [1,2]. Theories of behavior provide structured frameworks to explain how individuals make decisions and to identify factors that can be influenced to promote healthier behaviors. Despite broad recognition of their value, behavioral theories are still infrequently applied in the design of social media–based health interventions [3].

A recent systematic review of social media–based behavioral interventions in low- and middle-income countries (LMICs) found that, although most digital campaigns aimed to influence health behaviors, only a small fraction explicitly incorporated behavioral science theory or conceptual models in their design [3]. Of the 33 studies evaluated, only 5 used an established theory of behavior change, underscoring a major gap between behavioral science research and its application to digital health communication in LMICs [3].

Recognizing this weakness in the design of interventions, the World Health Organization (WHO) behavioral insights team has emphasized the importance of systematically applying behavioral science to accelerate progress toward global health goals, including vaccine uptake [4]. The WHO has called for public health programs to move beyond traditional information dissemination and awareness-raising and integrate behavioral, social, and contextual insights into intervention design to reflect the lived experience of individuals and communities. This shift recognizes that improving health outcomes often requires addressing the social and psychological drivers of behavior, not merely increasing awareness.

Building on this momentum, the WHO’s Behavioral Insights Unit has specifically highlighted the potential of social media campaigns informed by behavioral science to shape vaccine acceptance [5]. Using message framing strategies grounded in behavioral theory—such as gist versus verbatim representation—WHO’s recent campaigns demonstrated that applying behavioral insights can strengthen the persuasiveness of health messages delivered through digital channels. This experience provides an important foundation for designing social media interventions that go beyond traditional awareness-raising campaigns to target motivation and ability—two key drivers of behavior identified in the Fogg Behavior Model.

### 1.1. HPV Vaccination in Low- and Middle-Income Countries

Cervical cancer remains one of the leading causes of cancer-related deaths among women in low- and middle-income countries (LMICs) [6]. In Nigeria, an estimated 13,676 new cases of cervical cancer occurred in 2023, corresponding to an age-standardized incidence rate of 26.2 per 100,000 women. In the same year, approximately 7093 deaths were attributed to cervical cancer, yielding an age-standardized mortality rate of 14.3 per 100,000 women [7]. The disease is largely preventable through vaccination against the human papillomavirus (HPV), yet HPV vaccine coverage remains low in many settings [8]. Despite recent progress, vaccination campaigns in sub-Saharan Africa have faced logistical challenges, misinformation, and vaccine hesitancy. For instance, Kenya’s two-dose national campaign achieved only 33% coverage for the second dose [9], while Zambia and other countries have reported persistent difficulties in reaching caregivers who remain uncertain about vaccine safety and benefits [10].

In October 2023, the Government of Nigeria launched a nationwide school-based campaign introducing the single-dose human papillomavirus (HPV) vaccine for girls aged 9–14 years. The campaign was coordinated through the National Primary Health Care Development Agency and state education authorities. The vaccine was subsequently integrated into the routine immunization system to extend reach beyond time-limited campaigns and to improve access for girls who are not enrolled in school [11].

As HPV vaccination is incorporated into routine immunization platforms, maintaining high coverage introduces a new set of behavioral and implementation challenges. These challenges include sustaining caregiver engagement beyond scheduled campaigns, ensuring reliable access through primary health facilities, reaching girls who are not enrolled in school, and addressing misinformation and vaccine rejection. Recent evidence from Nigeria highlights a surge in HPV vaccine rejection driven by misinformation, fertility-related concerns, and residual distrust following the COVID-19 pandemic, underscoring the importance of effective communication strategies to support routine delivery [12,13].

Evidence from other low- and middle-income countries similarly shows that while school-based platforms efficiently reach enrolled adolescents, they systematically miss out-of-school girls and require complementary delivery and communication strategies to achieve equitable coverage [14]. More broadly, health system experiences across LMICs indicate that integrating HPV vaccination into national immunization programs introduces sustainability challenges that extend beyond logistics to include sociocultural acceptance, health worker capacity, and political commitment [15].

Together, this evidence highlights the need for interventions that address behavioral barriers alongside routine immunization delivery. Understanding how behavioral insights–informed interventions can complement existing service delivery platforms is therefore increasingly important. This study evaluates the additional impact of a behavioral insights–informed social media campaign amplified by local influencers, pharmacists, on HPV vaccine uptake during a period of routine immunization in Nigeria.

### 1.2. Role of Behavioral Insights and Digital Communication

Behavioral science offers promising tools for improving the design and delivery of public health interventions. The Fogg Behavior Model posits that behavior happens when three elements—motivation, ability, and a prompt—happen in the same moment [16]. In the context of a behavioral intervention, this implies that, by addressing drivers of and barriers to vaccination, and by providing timely prompts through social media and local influencers, caregivers can be encouraged to vaccinate their adolescents against HPV.

Social media use is rapidly expanding in Nigeria through Facebook and Instagram. In November 2025, Nigeria had an estimated 46,673,200 Facebook users, representing 19.5% of the national population [17]. This digital penetration provides a unique opportunity to complement traditional vaccination campaigns with behavioral insight-informed messaging that reaches caregivers directly in their online environments and in their physical environment through local pharmacists. Building on evidence from Bangladesh, where a behavioral insights–informed social media intervention significantly increased HPV vaccine uptake [18], the present study investigates whether a social media campaign enhanced the effectiveness of Nigeria’s routine HPV vaccination efforts.

### 1.3. Study Objective

This study evaluates the impact of a behavioral insights–informed social media campaign using local pharmacists as influencers implemented in 2025. This was a period during which HPV vaccination was being offered through routine immunization following two school-based mass vaccination campaigns conducted in 2023 and 2024. Using a quasi-experimental pre-test/post-test design with treatment and control areas, the study examines (1) the campaign’s impact on the HPV vaccination rate and (2) the mechanism through which the intervention had an impact on vaccination behavior.

## 2. Materials and Methods

### 2.1. Study Design

A pre-test/post-test quasi-experimental design with two rounds of cross-sectional surveys was implemented between April and November 2025 to assess the effect of a digital intervention using local pharmacists as influencers on HPV vaccination uptake during routine delivery of HPV vaccination through the national immunization program. The study was conducted in three Nigerian settings—Abuja (Federal Capital Territory), Adamawa, and Nasarawa—selected to represent urban, peri-urban, and semi-rural contexts with relatively high social media penetration.

Although states served as the sampling frame, treatment and control assignment were implemented at the level of local government areas (LGAs) to minimize contamination between exposed and unexposed populations. Treatment and control LGAs were defined prior to campaign launch and remained fixed throughout the study period. LGAs were purposively selected to allow meaningful geographic separation between treatment and control areas while maintaining broadly similar population profiles within states.

In each study state, treatment and control areas were selected within the same administrative and policy context to reduce the likelihood of systematic differences that were not related to the intervention. In Abuja, all Area Councils were designated as treatment areas except for the southern Abuja Municipal Area Council (AMAC) and northern Kuje, which served as control areas. In Nasarawa State, all LGAs except Akwanga and Nasarawa were designated as treatment areas. In Adamawa State, Yola North and Girei LGAs were designated as treatment areas, while all remaining LGAs served as controls. Abuja accounted for a larger share of participants due to the higher penetration of Meta platforms relative to the other study states.

### 2.2. Description of Intervention and Theory of Change

The Vaccination Initiative Targeting Adolescent Lives (VITAL) Project aimed to increase uptake of the HPV vaccine among girls aged 9–14 years in Nigeria through an integrated behavioral insights–informed approach. The intervention combined hyper-local social media outreach with direct pharmacist engagement in treatment areas within the Federal Capital Territory (FCT), Nasarawa, and Adamawa States. The underlying theory of change posits that repeated exposure to trustworthy information, delivered through credible local influencers, both online and in-person, increases a caregiver’s motivation and ability to vaccinate their adolescent girl.

Social media advertisements were designed to strengthen caregivers’ motivation and ability to vaccinate their child. Digital campaigns featured trusted local voices—including pharmacists as well as other local influencers—sharing accurate information about HPV vaccination. Content emphasized the benefits of vaccination for protecting a girl’s future health, reassurance about vaccine safety, and clear guidance on where and when vaccination could be obtained. Formats included caregiver testimonials, short videos, Nollywood drama, infographics, memes, polls, and live discussions designed to normalize vaccination and counter misinformation.

Pharmacist education focused on reinforcing these messages during routine caregiver interactions. Trained pharmacists at WellaHealth partner pharmacies addressed common caregiver concerns, provided information on eligibility and vaccination locations, and encouraged caregivers to take action during upcoming immunization sessions. Pharmacies displayed posters, brochures, and other branded materials that visually connected in-person interactions to the digital campaign. This dual approach was designed to build salience, trust, motivation, and ability to vaccinate.

Implementation was adaptive and data-driven. Early monitoring indicated that initial community-level geofencing was overly restrictive and limited campaign reach, prompting expansion to Local Government Area (LGA)–level targeting. Routine vaccination data also showed that most doses were administered during outreach events and Maternal, Newborn and Child Health (MNCH) Weeks, prompting intensified messaging around these high-traffic periods. By integrating continuous learning into campaign design, the project aligned behavioral insights with real-world service delivery dynamics to optimize impact.

As countries integrate HPV vaccination into routine immunization systems—while continuing to rely on school-based delivery to reach enrolled adolescents—maintaining vaccine uptake presents new behavioral and implementation challenges. These include sustaining caregiver engagement outside time-limited campaigns, reaching out-of-school girls, and ensuring consistent access through routine health services. Clarifying how behaviorally informed digital interventions can strengthen existing immunization delivery platforms has therefore become increasingly important. This study evaluates the additional impact of a digital behavior change intervention on HPV vaccination uptake during routine immunization in Nigeria.

### 2.3. Intervention Delivery, Geographic Targeting, and Platform Selection

The social media campaign was designed to support a quasi-experimental evaluation with clearly defined treatment and control areas. To minimize contamination and preserve internal validity, campaign exposure was assigned at the level of LGAs, which served as the lower administrative unit for geographic separation within states.

Platform selection was driven by this methodological requirement rather than by reach alone. Meta platforms (Facebook and Instagram) were selected because their advertising infrastructure allows targeting based on fine-grained geographic criteria, including LGAs, using a combination of device location signals (e.g., GPS, IP address, and network data). This enabled substantial differences in exposure intensity between treatment and control LGAs.

Other high-reach platforms were not used for primary campaign delivery because they could not meet this requirement. TikTok, although widely used in Nigeria, did not permit subnational geographic targeting at the LGA or state level during the study period. WhatsApp, while highly prevalent, relies on private or invitation-based group communication and does not support controlled broadcast advertising; it was therefore used only for internal coordination with participating pharmacists rather than for direct caregiver outreach.

Although perfect geographic isolation is not possible in digital advertising environments, any residual spillover across LGAs would bias estimated treatment effects toward the null. The observed differences between treatment and control areas therefore represent conservative estimates of campaign impact.

### 2.4. Participants and Recruitment

Caregivers of adolescent girls aged 9–17 years were recruited online through Facebook and Instagram advertisements. Although Nigeria’s HPV vaccination guidelines target girls aged 9–14 years, caregivers of girls aged 15–17 years were included to reflect real-world exposure to vaccination messaging and services during the transition from school-based campaigns to routine immunization. Including older adolescents also allowed assessment of potential spillover effects beyond the primary target age group. Robustness analyses restricted to caregivers of age-eligible girls (9–14 years) yielded substantively similar results and are presented in Supplementary Table S1.

Advertisements targeted caregivers aged 18 years and older residing in treatment and control local government areas (LGAs) within the study states. Recruitment procedures, eligibility criteria, and survey instruments were identical across states and across treatment and control LGAs. The advertisements described the study purpose and indicated that participants would receive mobile phone credit (approximately USD $0.41) upon survey completion. Respondents who identified themselves as caregivers of at least one adolescent girl were eligible to participate.

Upon clicking the advertisement, respondents were directed to a web-based survey hosted within the Facebook application. The survey opened with an introductory message explaining the study’s purpose, the voluntary nature of participation, and compensation details. Participants provided implied consent by clicking “Start” to initiate the survey. Surveys could be completed at participants’ convenience, with the option to pause and resume later.

Baseline data were collected from 9–17 April 2025, and follow-up data were collected from 10–17 November 2025. The study used independent cross-sectional samples at each time point. In total, 2504 caregivers completed the baseline survey and 3462 completed the follow-up survey. Surveys were administered in English and Hausa, the two predominant languages in the study areas.

Because recruitment relied on social media platforms, the sample likely overrepresents caregivers with internet access and higher digital literacy. While this limits generalizability to all caregivers of vaccine-eligible girls, it aligns with the delivery modality of the intervention and strengthens internal validity for estimating campaign effects among those exposed.

### 2.5. Data Collection and Measures

Baseline and follow-up surveys collected data on caregiver and adolescent demographics, adolescent HPV vaccination status, and key behavioral drivers of caregivers’ actions.

Primary outcome:

- *HPV vaccination status*—measured by asking: “Has the girl child in your care received an HPV vaccination?” Responses were coded as *Yes, No, or I don’t know*. Affirmative responses were classified as vaccinated.
  Secondary outcomes:
- *Motivation*—measured using the item: “Getting the girl who is in my care vaccinated against HPV is important to me.” Responses were captured on a five-point Likert scale (Strongly disagree–Strongly agree). Those selecting *Strongly agree* were classified as highly motivated.
- *Ability*—measured using two questions:
  1. “Getting the girl child in my care vaccinated against HPV is easy for me.”
  2. “I know where to get the HPV vaccine for the girl in my care.”
  Caregivers who selected *Strongly agree* were classified as having high perceived ability.
- *Knowledge*—assessed with the question: “At what age should the HPV vaccination ideally be given?” Respondents who answered 9–14 years were considered to have accurate knowledge.

All measures were self-reported. Surveys were administered in English or Hausa according to participant preference.

### 2.6. Statistical Analysis

Analyses were conducted using Stata 14. Descriptive statistics compared baseline characteristics between treatment and control areas using chi-squared tests for categorical variables. To estimate intervention effects, multivariate logistic regression models employing a difference-in-difference (DiD) approach were used.

The general model was:

$$\mathrm{logit}(Y_{it})=\beta_{0}+\beta_{1}{\mathrm{Treatment}}_{i}+\beta_{2}{\mathrm{Post}}_{t}+\beta_{3}({\mathrm{Treatment}}_{i}\times {\mathrm{Post}}_{t})+X_{it}\gamma +\varepsilon_{it}$$

where $Y_{it}$ is the outcome for respondent *i* at time *t*; Treatment indicates assignment to the treatment area; Post denotes follow-up; and $X_{it}$ represents covariates including caregiver gender, age, education, adolescent age, interview language, and state.

The coefficient β_3_ estimates the additional differential change associated with the intervention beyond the national campaign, adjusting for covariates. Adjusted odds ratios (aORs) and 95% confidence intervals were reported. Average marginal effects of the treatment × post interaction were computed to derive adjusted predicted probabilities of vaccination, holding covariates at their observed values, to improve interpretability.

In parallel, DiD models were estimated for motivation, ability, and knowledge outcomes to examine intervention effects on behavioral mechanisms consistent with the Fogg Behavior Model.

Although the national HPV vaccination guidelines target girls aged 9–14 years, caregivers of girls aged 15–17 years (approximately 11% of the sample) were included in baseline and follow-up surveys. To assess robustness of findings, we also conducted analyses restricted to caregivers of 9–14-year-olds. This analysis yielded substantively similar results to those obtained using the full sample.

### 2.7. Ethical Considerations

Participation was voluntary, and electronic informed consent was obtained from all respondents before survey initiation. No personally identifiable information (PII) was collected. The study protocol, including the consent process, was reviewed and approved by the National Health Research Ethics Committee of Nigeria (NHREC) on 15 July 2024 under protocol number NHREC/01/01/2007–15/07/2024. Its extension was approved on 1 September 2025 under protocol number NHREC/01/01/2007-29/08/2025. All research procedures adhered to the ethical principles of the Declaration of Helsinki.

## 3. Results

### 3.1. Sample Characteristics

A total of 5932 caregivers of adolescent girls aged 9–17 years participated in the repeated cross-sectional baseline and follow-up surveys conducted between April and November 2025. Of these, 3118 respondents were from the treatment area, where the behavioral insights–informed social media campaign was conducted and pharmacists were trained to counsel caregivers on HPV vaccination. A total of 2814 caregivers were from the control area, where routine HPV vaccination services were provided but the social media intervention and pharmacists’ counseling were not.

Table 1 summarizes the socio-demographic characteristics of caregivers and adolescents in treatment and control areas.

#### 3.1.1. Gender of Caregiver

Female caregivers constituted roughly half of the total sample, with a slightly higher proportion in the treatment area (49.9%) compared to the control area (46.9%) (*p* = 0.027).

#### 3.1.2. Age of Caregiver

The majority of caregivers were aged 18–39 years, consistent with the typical age range of caregivers of adolescent girls. Caregivers aged 18–29 comprised 50.8% of the treatment group and 50.4% of the control group. The proportion of older caregivers, those aged 40 or older, was slightly lower in the treatment area (10.8%) than in the control area (13.3%) (*p* = 0.007).

#### 3.1.3. Education of Caregiver

Educational attainment was relatively high across both areas. Approximately one-third of caregivers held a Higher National Diploma (HND) or bachelor’s degree (34.5% in the treatment area; 33.1% in the control area), while around one in ten reported having a postgraduate qualification (11.4% and 12.1%, respectively). A small proportion of caregivers had no formal education (6.1% in the treatment area; 5.9% in the control area). Differences in education were not statistically significant (*p* = 0.142), suggesting comparable levels of educational attainment between areas.

#### 3.1.4. Age of Adolescent Girl

Girls aged 9–11 constituted the largest group in both treatment and control areas (about 60.2% of girls in the treatment area and 56.5% in the control area), followed by those aged 12–14 (28.9% and 31.5%, respectively) and 15–17 (11.0% and 12.0%, respectively). The difference in the age distribution of adolescents in treatment and control areas was statistically significant (*p* = 0.013).

#### 3.1.5. Language of Interview

Interviews were conducted in English or Hausa, reflecting regional linguistic diversity. Just over half of the interviews were conducted in English (52.7% in the treatment area; 51.9% in the control area), while the remainder were conducted in Hausa. The similarity across groups (*p* = 0.538) suggests that the language of the interview was not systematically related to treatment status.

#### 3.1.6. State of Residence

Most caregivers were from Abuja, reflecting its higher population density and online reach within the sampling frame. A larger proportion of caregivers in the control area were from Abuja (89.7%) compared to the treatment area (80.1%), while the treatment area had higher representation from Adamawa (11.3% versus 6.4%) and Nasarawa (8.6% versus 3.9%) (*p* < 0.001).

#### 3.1.7. Summary

While the socio-demographic characteristics of the treatment and control groups were comparable, there were several statistically significant differences. These differences were sufficient to merit the inclusion of these variables in subsequent multivariate analysis to estimate the impact of the social media campaign.

### 3.2. Caregiver Characteristics and Their Relationship to HPV Vaccination at Baseline

Table 2 presents caregiver and adolescent characteristics at baseline (Column 1) and the relationship between these characteristics and adolescent HPV vaccination (Column 2). The distributions of caregiver characteristics at baseline were broadly consistent with those observed in Table 1 and reflect comparability between the treatment and control samples at baseline. Column 2 shows the associations between caregiver characteristics and vaccination, underscoring the importance of controlling for these factors in multivariate analyses.

Overall, 54.7% of adolescent girls were reported to have received the HPV vaccine at baseline. Vaccination rates varied significantly across caregiver characteristics.

#### 3.2.1. Gender of Caregiver

Vaccination coverage was slightly higher among adolescents with female caregivers (57.0%) than among those with male caregivers (53.0%) (*p* = 0.046). This suggests that female caregivers may have been more proactive in supporting their daughters’ vaccination, a pattern observed in other LMIC settings.

#### 3.2.2. Age of Caregiver

A caregiver’s age was strongly associated with adolescent vaccination (*p* < 0.001). The proportion of vaccinated adolescents declined steadily with caregivers’ age—from 58.8% among caregivers aged 18–29 years to 52.9% among those aged 30–39 years, and 44.7% among caregivers aged 40 years or older. Younger caregivers may be more receptive to new vaccines or more likely to engage with health messages on digital platforms.

#### 3.2.3. Education of Caregiver

Vaccination rates varied significantly by education level (*p* < 0.001). As shown by previous social media surveys in Nigeria, the highest HPV vaccination rate (80.2%) was observed among caregivers with no formal education, while those with primary or secondary education reported lower vaccine uptake (65.2% and 56.3%, respectively). Vaccination prevalence declined further among caregivers with postsecondary education, ranging from 50.1% among those with OND to 51.0% among those with HND/BSc, and 50.2% among those with postgraduate degrees.

This inverse association may reflect contextual factors such as varying levels of exposure to the government’s school-based vaccination campaigns. A similar pattern has been observed in other social media–based surveys in Nigeria. A recent study of 4830 caregivers of adolescent girls found that caregivers with no formal education had significantly higher exposure to HPV vaccine campaign messages and were three times more likely to have vaccinated their daughters than those with higher education levels [11]. These findings suggest that educational disparities in exposure to and responsiveness to vaccination messaging may partly explain the counterintuitive association observed in the present study.

#### 3.2.4. Age of Adolescent Girl

The age of the adolescent girl was also significantly associated with vaccination status (*p* < 0.001). The youngest group (ages 9–11) had the highest vaccination prevalence (60.7%), followed by those aged 12–14 years (50.8%). The oldest group (15–17 years) had substantially lower vaccination rates (35.2%), possibly reflecting reduced parental engagement or school attendance among older adolescents. These findings suggest that the effects of the government’s HPV vaccination campaign extended beyond adolescents in the immediate target age group, consistent with spillover benefits during the transition to routine immunization.

#### 3.2.5. Language of Interview

Vaccination rates differed modestly by interview language (*p* = 0.017). Adolescents whose caregivers were interviewed in Hausa reported higher vaccination coverage (57.5%) than those whose caregivers were interviewed in English (52.7%), suggesting that caregivers reached through local-language communication channels may have been more responsive to vaccination messaging.

#### 3.2.6. State of Residence

Significant differences were observed across states (*p* < 0.001). Caregivers residing in Abuja reported the highest vaccination coverage (56.4%), compared to 51.2% in Nasarawa and 35.4% in Adamawa. These patterns may reflect differences in campaign implementation intensity or access to vaccination services across geographic areas.

#### 3.2.7. Summary

Taken together, these findings demonstrate that caregiver and adolescent characteristics—particularly caregiver age, education, and state of residence—were significantly associated with HPV vaccination at baseline. This strengthened the case for including these as covariates in subsequent multivariate analyses to adjust for potential confounding effects and to estimate the differential change in vaccination associated with the intervention.

### 3.3. Change in HPV Vaccination Rates: Unadjusted Analysis

Table 3 presents the unadjusted change in HPV vaccination rates between baseline and follow-up in the treatment and control areas. This bivariate analysis illustrates the difference-in-difference (DiD) approach later formalized in the multivariate logistic regression models. Presenting these unadjusted changes provides transparency regarding baseline differences and clarifies how covariate adjustment in subsequent models affects the estimated intervention effect.

At baseline, 55.6 percent of adolescent girls in the treatment area were reported to have received the HPV vaccine compared to 51.6 percent in the control area. Vaccination coverage was similar at baseline between treatment and control areas (*p* > 0.05). After the intervention, coverage increased to 62.3% in the treatment area and 56.2% in the control area.

The absolute increase in vaccination between baseline and follow-up was 6.7 percentage points in the treatment area and 4.6 percentage points in the control area. The unadjusted difference-in-difference of +2.1 percentage points indicates that HPV vaccination increased more in the treatment area than in the control area during the study period.

Although modest in magnitude, this difference provides an initial indication that the intervention contributed to higher vaccination uptake. The subsequent multivariate analysis examines whether this additional effect remains statistically significant after adjusting for differences in caregiver and adolescent characteristics between treatment and control groups. As this analysis is unadjusted and descriptive, confidence intervals are not reported; formal statistical inference is presented in the adjusted regression models that follow.

### 3.4. Multivariate Logistic Regression Results: Impact of the Behavioral Insights–Informed Social Media Intervention

Table 4 presents findings from multivariate logistic regression models estimating the adjusted odds of HPV vaccination, controlling for caregiver and adolescent characteristics. All models controlled for state of residence, ensuring that baseline differences in vaccination levels across Abuja, Adamawa, and Nasarawa did not confound the estimated association between the intervention and HPV vaccine uptake. Model 1 shows the main effects of socio-demographic variables and time (baseline versus follow-up), while Model 2 introduces the interaction term (Treatment × Follow-up) to capture the differential change in vaccination associated with the intervention.

### 3.5. Main Effects Model (Model 1)

After adjusting for socio-demographic characteristics, adolescent girls in the treatment area had higher odds of being vaccinated than adolescent girls in the control area (aOR = 1.21, 95% CI: 1.07–1.37). The odds of being vaccinated were also higher at follow-up than at baseline (aOR = 1.22, 95% CI: 1.08–1.38).

Several characteristics of caregivers and adolescents were significantly associated with HPV vaccination. A child whose caregiver was female had a higher odds of being vaccinated than a child whose caregiver was a male (aOR = 1.19, 95% CI: 1.07–1.32). Caregivers aged 18–29 had a higher odds ratio of being vaccinated (aOR = 1.31, 95% CI: 1.09–1.57) compared to caregivers aged 40 years or older.

Caregivers’ education was inversely associated with vaccination. Compared with adolescents whose caregivers had no formal education, adolescents whose caregivers had primary, secondary, or postsecondary education had significantly lower odds of being vaccinated. For example, adolescents whose caregivers had HND/BSc were less than half as likely to be vaccinated than those with no formal education (aOR = 0.43, 95% CI: 0.33–0.56). These findings are consistent with results from earlier research on HPV vaccination in Nigeria, which found that less educated caregivers were more frequently exposed to and more responsive to HPV vaccine messaging on social media [11].

The adolescent girl’s age was strongly associated with vaccination. Girls aged 9–11 years had more than twice the odds of being vaccinated compared to those aged 15–17 years (aOR = 2.31, 95% CI: 1.94–2.76), while those aged 12–14 years also had higher odds (aOR = 1.50, 95% CI: 1.25–1.81) of being vaccinated. Caregivers who responded to the questionnaire in Hausa reported higher odds of their child being vaccinated than those who responded to the questionnaire in English (aOR = 1.38, 95% CI: 1.21–1.51). State-level differences in vaccination rates were observed: the odds of a child being vaccinated were significantly lower in Adamawa (aOR = 0.66, 95% CI: 0.54–0.79) compared with Abuja, while differences between Nasarawa and Abuja were not statistically significant.

### 3.6. Interaction Effects Model (Model 2)

When the interaction term between treatment and follow-up was included, the model revealed a statistically significant differential change associated with the intervention. The interaction term (Treatment × Follow-up) had an adjusted odds ratio of 1.48 (95% CI: 1.14–1.92), indicating that the odds of HPV vaccination increased by nearly 50 percent in the treatment area relative to the control area after implementation of the intervention, controlling for all other variables.

This result is consistent with the intervention having contributed to higher HPV vaccine uptake in the treatment area. The finding aligns with the unadjusted difference-in-difference estimate presented in Table 3 and suggests that exposure to the intervention was associated with higher vaccination coverage, under the assumption of the difference-in-difference design.

### 3.7. Adjusted Predicted Probabilities of HPV Vaccination

Table 5 presents adjusted predicted probabilities of HPV vaccination from the multivariate logistic regression model. These probabilities were derived using average marginal effects estimation to translate the odds ratios in Table 4 into more interpretable changes in vaccination likelihood. The adjusted estimates account for differences in caregiver and adolescent characteristics between treatment and control areas, providing a clearer assessment of the differential change in vaccination associated with the intervention.

At baseline, the predicted probability of HPV vaccination was 0.5545 in the treatment area and 0.5656 in the control area, indicating similar initial vaccination levels before implementation of the intervention. Following the national campaign and the introduction of the behavioral insights–informed social media campaign in which pharmacists served as influencers, the predicted probability of vaccination in the treatment area increased to 0.6354, while in the control area it remained essentially unchanged at 0.5570.

The adjusted increase in HPV vaccination between baseline and follow-up was 8.1 percentage points in the treatment area, compared with a decline of 0.9 percentage points in the control area.

The adjusted probability of HPV vaccination in the treatment area increased from 0.5545 at baseline to 0.6354 at follow-up, while remaining largely unchanged in the control area (0.5656 to 0.5570). The resulting adjusted difference-in-difference estimate was 8.9 percentage points (95% CI: 3.0–14.9), suggesting that the intervention was associated with an increase in the probability of adolescent HPV vaccination of approximately nine percentage points. This represents a substantially larger estimate than the unadjusted 2.1-percentage-point difference reported in Table 3. The divergence between the two estimates is primarily attributable to baseline compositional differences between treatment and control area samples. In particular, the control area had a higher proportion of respondents from Abuja (89.7% versus 80.1% in the treatment area), where baseline vaccination coverage was highest. This imbalance suppressed the unadjusted estimate because the control area benefited disproportionately from higher-coverage Abuja respondents. Once the multivariate model adjusted for state of residence, caregiver age, education, and other covariates, the differential in vaccination trends between treatment and control areas became more apparent. These findings indicate that, after controlling for socio-demographic differences, the intervention was associated with a meaningful increase in HPV vaccination uptake, under the assumptions of the difference-in-difference design.

### 3.8. Mechanisms of Change: Effects of the Intervention on Motivation and Ability

Table 6 presents multivariate logistic regression results assessing how the intervention was associated with changes in motivation and ability, as hypothesized by the Fogg Behavior Model, to drive vaccination behavior. The models include interaction terms (Treatment × Follow-up) to identify whether changes in motivation, ability, or knowledge differed between the treatment and control areas after implementation of the intervention. All models controlled for caregiver and adolescent socio-demographic characteristics, including caregiver age, gender, education, language of interview, adolescent age, and state of residence. For simplicity, these control variables are not shown in the table.

### 3.9. Motivation

Column 1 shows that caregivers in the treatment area were more likely than those in the control area to report that getting their child vaccinated was important to them. The interaction term (Treatment × Follow-up) was statistically significant (aOR = 1.31, 95% CI: 1.00–1.70), indicating that motivation to vaccinate increased at a higher rate in the treatment area than in the control area between baseline and follow-up surveys. This finding suggests that the intervention was associated with higher caregiver motivation to vaccinate their child.

### 3.10. Ability

Columns 2 and 3 show that the intervention was also associated with a positive and statistically significant differential change in caregivers’ ability to vaccinate their child. The interaction term was significant for both measures of ability: knowing where to get the child vaccinated (aOR = 1.38, 95% CI: 1.07–1.79) and that vaccination was easy to obtain (aOR = 1.59, 95% CI: 1.22–2.06). These findings suggest that the intervention was associated with reduced logistical and psychological barriers to vaccination—key elements of the “ability” construct in the Fogg Behavior Model.

### 3.11. Knowledge

By contrast, no statistically significant change in caregivers’ knowledge of the correct age for HPV vaccination was associated with the intervention (aOR = 0.87, 95% CI: 0.67–1.13). This suggests that the rate of change in factual knowledge about HPV vaccination did not differ between treatment and control groups.

### 3.12. Summary

Together, these findings provide evidence consistent with the intervention having contributed to a higher HPV vaccination rate in the treatment area. Consistent with the Fogg Behavior Model, the observed changes suggest that the intervention operated primarily through behavioral, rather than informational, pathways.

## 4. Discussion

The results of this study are consistent with the intervention having contributed to higher HPV vaccination among adolescent girls in treatment relative to control areas. After controlling for socio-demographic differences between treatment and control areas, adjusted difference-in-difference estimates suggest that the intervention was associated with an increase in the probability of vaccination by approximately nine percentage points. This estimate is meaningful in magnitude and is consistent with the intervention having contributed to higher HPV vaccination uptake during a period when vaccination was being delivered through routine immunization following two earlier school-based mass campaigns.

### 4.1. Behavioral Pathways of Impact

Analysis of motivation and ability outcomes provides insight into the mechanisms through which the intervention may have achieved its effect. The higher rates of motivation and perceived ability observed in treatment areas relative to control areas are consistent with the behavioral pathway proposed by the Fogg Behavior Model, which states that behavior occurs when sufficient motivation and ability are present and an effective prompt triggers action. Increases in vaccination coincided with improvements in caregivers’ motivation and perceived ability, but not with gains in factual knowledge, indicating that the intervention acted mainly on behavioral determinants rather than informational gaps.

These findings suggest that the intervention’s behavioral framing may have contributed to the observed differences. Content emphasized community approval, emotional rewards associated with protecting a child’s future, and the practical ease of vaccination.

### 4.2. Comparison with Other Evidence

The magnitude and direction of the association observed in Nigeria are consistent with findings from Bangladesh, where a behavioral insights–informed social media campaign implemented alongside the national vaccine introduction was associated with higher HPV vaccination beyond the effects of the national program. The consistency of findings across two distinct LMIC settings suggests that behaviorally informed digital communication strategies may effectively complement school-based vaccination delivery systems.

The inverse association between caregiver education and HPV vaccination observed here is consistent with earlier evidence from Nigeria [11], which showed that caregivers with lower levels of formal education were more likely to encounter and respond to vaccine-related content disseminated through social media. Together, these results underscore the need to tailor digital communication not only to reach underserved groups but also to engage more educated caregivers, who may be more skeptical of vaccination campaigns.

### 4.3. Implications for Vaccine Communication Strategy

These findings have several practical implications. First, they highlight the value of applying behavioral science frameworks such as the Fogg Behavior Model to guide the design of public health communication. Second, they are consistent with targeted behavioral interventions being capable of shifting the psychological determinants of vaccination —motivation and ability. Finally, these findings suggest that integrating behavioral insights-informed campaigns into national vaccination programs may help sustain demand for immunization following mass vaccination campaigns.

### 4.4. Limitations

As in other social media–based studies, this analysis is subject to limitations. Recruitment through Facebook and Instagram may have excluded caregivers without internet or social media access, potentially overrepresenting urban and younger caregivers and limiting the generalizability of findings to more connected populations. Vaccination status was caregiver-reported, which may introduce recall or social desirability bias. While concerns about social desirability bias among less-educated respondents are valid, the broader literature suggests that socioeconomic status is only modestly associated with social desirability bias, and findings are often mixed.

Cross-sectional survey data provide only a snapshot of caregiver behaviors. A longitudinal design would be better suited to capture temporal changes and provide deeper insights into how motivation, ability, and vaccination behaviors evolve over time. Despite these limitations, the consistency of results across motivation, ability, and vaccination outcomes, and the large sample size drawn from multiple states, strengthens confidence in the validity of the findings.

This study relies on a difference-in-difference analytical approach, which assumes that, in the absence of the intervention, trends in HPV vaccination would have evolved similarly in treatment and control areas (the parallel trends assumption). However, no pre-intervention trend data are available to formally assess whether vaccination trends were evolving similarly in the two groups before implementation, which limits the ability to verify this assumption empirically. In addition, the study uses repeated independent cross-sectional surveys rather than longitudinal follow-up of the same caregivers, and treatment and control areas were purposively selected rather than randomized. These design features constrain causal inference: while the DiD design differences out unobserved time-invariant differences between areas, it cannot fully account for unobserved time-varying factors that may have differentially affected vaccination uptake during the study period. The findings should therefore be interpreted as evidence of an association consistent with a combined intervention effect under the stated DiD assumptions, rather than as firm causal proof. Nonetheless, the inclusion of repeated cross-sectional data, adjustment for observed caregiver and adolescent characteristics, and the consistency of findings across multiple outcomes reduce the likelihood that the observed associations are driven entirely by confounding.

### 4.5. Policy and Research Implications

The findings of this study are consistent with behavioral insights-informed interventions playing a meaningful role in strengthening national immunization programs. In contexts such as Nigeria—where social media use is widespread and health communication resources are limited—digital campaigns that incorporate behavioral insights may offer a scalable and cost-effective complement to traditional vaccination outreach. By addressing motivation and ability-related barriers rather than relying solely on information dissemination, such interventions help sustain demand for vaccines and counteract hesitancy driven by misinformation or apathy.

As digital platforms continue to expand across sub-Saharan Africa, the integration of behavioral science principles into public health communication offers a powerful tool for achieving high vaccine coverage and advancing the goals of cervical cancer prevention in low- and middle-income countries.

## 5. Conclusions

A behavioral insights–informed social media campaign in which pharmacists served as local influencers was associated with vaccine uptake when integrated with national immunization efforts. In Nigeria, the intervention—which targeted caregivers’ motivation and ability—was associated with higher HPV vaccination rates during a period of routine immunization following two nationwide school-based mass vaccination campaigns. The intervention was associated with an increase in HPV vaccination of approximately nine percentage points among adolescent girls.

These findings are consistent with the potential of behaviorally grounded digital communication strategies to complement traditional vaccine delivery platforms, sustain demand for immunization beyond mass campaigns, and address behavioral barriers that limit uptake. As social media use continues to expand across sub-Saharan Africa, integrating behavioral insights into public health interventions offers a scalable and cost-effective approach to strengthening immunization programs and advancing cervical cancer prevention in low- and middle-income countries.

## Figures and Tables

**Table 1:** Caregiver characteristics in treatment and control areas.

Variables	(1) Treatment % (n = 3118)	(2) Control (n = 2814)	*p* Value
Gender of caregiver			
Male	50.1	53.1	0.027
Female	49.9	46.9	
Age of caregiver			
18–29	50.8	50.4	0.007
30–39	38.5	36.3	
40 and older	10.8	13.3	
Education of caregiver			
None	6.1	5.9	0.142
Primary School Certificate	8.7	7.8	
SSCE/GCE	24.0	26.8	
OND	15.3	14.4	
HND/BSc	34.5	33.1	
Postgraduate degree	11.4	12.1	
Age of adolescent girl			
9–11	60.2	56.5	0.013
12–14	28.9	31.5	
15–17	11.0	12.0	
Language of Interview			
English	52.7	51.9	0.538
Hausa	47.3	48.1	
State			
Abuja	80.1	89.7	<0.001
Adamawa	11.3	6.4	
Nasarawa	8.6	3.9	
Total	100%	100%	

**Table 2:** Caregiver characteristics and their relationship with HPV vaccination at baseline.

Variables	(1) Caregiver Characteristics at Baseline % (n = 2504)	(2) % of Adolescents Vaccinated at Baseline (n = 1369)	*p* Value
Gender of caregiver			
Male	49.9	53.0	0.046
Female	50.1	57.0	
Age of caregiver			
18–29	48.8	58.8	<0.001
30–39	38.0	52.9	
40 and older	13.2	44.7	
Education of caregiver			
None	5.0	80.2	<0.001
Primary School Certificate	8.3	65.2	
SSCE/GCE	23.5	56.3	
OND	13.5	50.1	
HND/BSc	37.6	51.0	
Postgraduate degree	12.1	50.2	
Age of adolescent girl			
9–11	56.2	60.7	<0.001
12–14	32.9	50.8	
15–17	10.9	35.2	
Language of Interview			
English	58.5	52.7	0.017
Hausa	41.5	57.5	
**State**			
Abuja	87.9	56.4	<0.001
Adamawa	7.0	35.4	
Nasarawa	5.2	51.2	
Total	100%	54.7%	

**Table 3:** Change in the HPV vaccination rate, unadjusted.

Variables	(1) Baseline (n = 2504)	(2) Follow-Up (n = 3462)	(3) Change
Treatment	55.6	62.3	+6.7
Control	51.6	56.2	+4.6
Difference-in-difference			+2.1

Note: Baseline and follow-up samples are independent cross-sections.

**Table 4:** Adjusted odds of HPV vaccination showing effects of the social media intervention on HPV vaccination (difference-in-difference model).

Variables	(1) Adjusted Odds of Vaccination (Pooled Data) (n = 5774)	(2) Adjusted Odds of Vaccination (Pooled Data, Showing Treatment Effects) (n = 5774)
Treatment status		
Treatment	1.21 (1.07–1.37)	0.95 (0.78–1.17)
Control	Ref	Ref
Survey round		
Follow-up	1.22 (1.08–1.38)	0.96 (0.79–1.18)
Baseline	Ref	Ref
Treatment × Follow-up	-	1.48 (1.14–1.92)
Gender of caregiver		
Male	Ref	Ref
Female	1.19 (1.07–1.32)	1.19 (1.07–1.33)
Age of caregiver		
18–29	1.31 (1.09–1.57)	1.32 (1.10–1.59)
30–39	1.16 (0.98–1.38)	1.15 (0.96–1.39)
40 and older	Ref	Ref
Education of caregiver		
None	Ref	Ref
Primary School Certificate	0.71 (0.52–0.98)	0.71 (0.53–0.99)
SSCE/GCE	0.49 (0.37–0.64)	0.49 (0.38–0.65)
OND	0.49 (0.36–0.65)	0.49 (0.37–0.65)
HND/BSc	0.43 (0.33–0.56)	0.43 (0.33–0.56)
Postgraduate degree	0.52 (0.38–0.70)	0.52 (0.38–0.69)
Age of adolescent girl		
9–11	2.31 (1.94–2.76)	2.31 (1.94–2.76)
12–14	1.50 (1.25–1.81)	1.50 (1.25–1.82)
15–17	Ref	Ref
Language of Interview		
English	Ref	Ref
Hausa	1.38 (1.21–1.51)	1.38 (1.24–1.54)
State		
Abuja	Ref	Ref
Adamawa	0.66 (0.54–0.79)	0.62 (0.52–0.76)
Nasarawa	0.85 (0.68–1.07)	0.83 (0.66–1.04)
Pseudo R-squared	4.12%	4.24%

Note: Odds ratios (OR) with 95% confidence intervals from logistic regression models. Model 1 shows adjusted associations using pooled baseline and follow-up data. Model 2 presents the difference-in-difference specification including treatment status, follow-up period, and their interaction. The interaction term (Treatment × Follow-up) represents the estimated effect of the social media intervention. Reference categories are: control group, baseline survey round, male caregiver, caregiver age ≥40 years, no formal education, adolescent girl age 15–17 years, English interview language, and Abuja state.

**Table 5:** Change in HPV vaccination: adjusted predicted probabilities (standard errors in parentheses).

Variables	(1) Baseline	(2) Follow-Up	(3) Change
Treatment	0.5545 (0.011)	0.6354 (0.014)	+0.0809
Control	0.5656 (0.021)	0.5570 (0.011)	−0.0086
Adjusted difference-in-difference			0.0895 (95% CI: 0.030–0.149)

Note: Values are adjusted predicted probabilities from multivariate logistic regression models; standard errors are shown in parentheses. The adjusted difference-in-difference and 95% confidence interval are derived from model-based linear contrasts of predicted probabilities.

**Table 6:** Adjusted odds showing effects of social media intervention on motivation and ability (difference-in-difference models).

Variables	(1) Motivation: Importance of Getting Child Vaccinated (n = 5774)	(2) Ability: Knowing Where to Get Child Vaccinated (n = 5774)	(3) Ability: Getting the Child Vaccinated is Easy (n = 5774)	(4) Knowledge: Reported Correct Age of HPV Vaccination (n = 5774)
Control	Ref	Ref	Ref	Ref
Treatment	1.18 (0.96–1.44)	1.17 (0.95–1.43)	1.12 (0.92–1.37)	1.44 (1.17–1.76)
Baseline	Ref	Ref	Ref	Ref
Follow-up	1.30 (1.07–1.59)	1.37 (1.12–1.68)	1.35 (1.11–1.65)	1.76 (1.44–2.15)
Interaction: Treatment × Follow-up	1.31 (1.00–1.70)	1.38 (1.07–1.79)	1.59 (1.22–2.06)	0.87 (0.67–1.13)
Pseudo R-squared	3.35%	5.17%	4.57%	7.90%

## Data Availability

Data supporting the findings of this study are available upon reasonable request from the corresponding author.

## Supplementary Materials

The following supporting information can be downloaded at: https://www.mdpi.com/article/10.3390/vaccines14040328/s1, Table S1: Adjusted odds of HPV vaccination showing effects of the social media intervention on HPV vaccination (difference-in-difference model).
