# Supplementary material for: Evaluating a Behavioral Insights–Informed Social Media Campaign to Increase HPV Vaccination During Routine Immunization in Nigeria
Source: Vaccines (Basel). 2026 Apr 7;14(4):328. doi: 10.3390/vaccines14040328 (PMC13120092; doi:10.3390/vaccines14040328)
Supplement: Supplementary file 1 [file vaccines-14-00328-s001.zip › vaccines-4086825-supplementary.pdf]

Table S1. Adjusted odds of HPV vaccination showing effects of the social media intervention on HPV vaccination (difference-in-difference model)

**Variables**	**(1)**<br>**Adjusted odds of vaccination (pooled data)**<br>(n=5117)	**(2)**<br>**Adjusted odds of vaccination (pooled data, showing treatment effects)**<br>(n=5117)
**Treatment**	1.24 (1.09 – 1.42)	0.89 (0.72 – 1.11)
**Control**	Ref	Ref
**Follow-up**	1.19 (1.04 – 1.35)	0.86 (0.69 – 1.07)
**Baseline**	Ref	Ref
**Treatment * Follow-up**	-	1.72 (1.30 – 2.27)
**Gender of caregiver**		
Male	Ref	Ref
Female	1.23 (1.10 – 1.39)	1.23 (1.09 – 1.38)
**Age of caregiver**		
18-29	1.48 (1.22 – 1.81)	1.52 (1.25 – 1.85)
30-39	1.29 (1.06 – 1.56)	1.28 (1.05 – 1.56)
40 and older	Ref	Ref
**Education of caregiver**		
None	Ref	Ref
Primary School Certificate	0.68 (0.49 – 0.95)	0.70 (0.50 – 0.97)
SSCE/GCE	0.46 (0.34 – 0.61)	0.47 (0.35 – 0.62)
OND	0.47 (0.34 – 0.64)	0.47 (0.35 – 0.64)
HND/BSc	0.41 (0.31 – 0.55)	0.41 (0.31 – 0.55)
Postgraduate degree	0.52 (0.38 – 0.72)	0.51 (0.37 – 0.71)
**Age of adolescent girl**		
9-11	1.53 (1.35 – 1.72)	1.52 (1.35 – 1.72)
12-14	Ref	Ref
15-17	-	-
**Language of Interview**		
English	Ref	Ref
Hausa	1.37 (1.22–1.54)	1.42 (1.26 – 1.59)
**State**		
Abuja	Ref	Ref

Adamawa	0.70 (0.57 – 0.86)	0.65 (0.53 – 0.80)
Nasarawa	0.85 (0.66 – 1.08)	0.82 (0.64 – 1.05)
**Pseudo R-squared**	3.54%	3.76%

Note: The analysis in this table is limited to caregivers whose adolescents are ages 9-14
